# Mechanical Power and Driving Pressure: Mechanisms of Lung Injury, Markers of Pathophysiology, or Therapeutic Targets?

**DOI:** 10.3390/jcm15010079

**Published:** 2025-12-22

**Authors:** Gary Frank Nieman, Joaquin Araos, Joshua Satalin, Penny Andrews, Nader Habashi

**Affiliations:** 1 Department of Surgery, Upstate Medical University, Syracuse, NY 13210, USA; 2 Department of Clinical Sciences, College of Veterinary Medicine, Cornell University, Ithaca, NY 14853, USA; 3 Department of Critical Care, R Adams Cowley Shock Trauma Center, University of Maryland, Baltimore, MD 21201, USA

**Keywords:** ARDS, VILI, mechanical power, driving pressure, lung recruitment, EELV

## Abstract

Acute respiratory distress syndrome (ARDS) causes heterogeneous injury, with normal, unstable, and edematous tissue distributed throughout the lung. Although positive pressure ventilation initially reduced ARDS-related mortality, it became clear that the ventilator can be a double-edged sword and, if set improperly, can worsen outcomes. This uneven pathology makes the lung vulnerable to secondary ventilator-induced lung injury (VILI). In 2000, evidence showed that lowering tidal volume (V_T_) and airway pressure significantly reduced mortality in patients with ARDS, suggesting that this reduction led to less overdistension of healthy lung tissue. Including respiratory system compliance (C_RS_) in the calculation. It was shown that low driving pressure (ΔP = V_T_/C_RS_) was more strongly associated with survival than low V_T_ alone. This idea was further extended into measuring the mechanical power delivered to the respiratory system: MP_rs_ = RR × ΔV^2^∙[1/2∙EL_rs_ + RR∙(1 + I:E)/60∙I:E∙R_aw_] + ΔV∙PEEP, where EL_rs_ is elastance, I:E is inspiratory:expiratory ratio, R_aw_ is airway resistance, and RR is respiratory rate. This measure helps identify when the lung is at risk of VILI. However, a recent study found no direct causal link between MP_RS_ and mortality; rather, it showed that MP_RS_, normalized to C_RS_ or end-expiratory lung volume (EELV), was independently associated with outcomes. This indicates that lung size and underlying pathophysiology—rather than ΔP or MP_RS_ alone—are critical determinants of VILI risk. Reopening collapsed lung tissue would increase C_RS_ and decrease E_RS_, thereby lowering ΔP or MP_RS_ at any given V_T_, R_aw_, PEEP, I:E, or RR setting. Consequently, the focus should shift from simply adjusting the ventilator to normalize C_RS_ and EELV that reduce ΔP or MP_RS_ at higher ventilator settings.

What does this work add that is new?

- This perspective study confirms that, for patients with ARDS, the current strategy to reduce the mechanical power of the respiratory system (MP_rs_) to minimize ventilator-induced lung injury (VILI) involves lowering ventilator settings, such as tidal volume, peak and end-expiratory pressures, or respiratory rate.
- New evidence indicates that, to measure VILI effectively, MP_rs_ must be normalized to respiratory system compliance (C_RS_) or end-expiratory lung volume (EELV), suggesting that lung pathophysiology and morphology are as crucial as elevated MP_rs_ in causing VILI.
- Therefore, a better goal for reducing MP_rs_ is to focus on gradually re-expand the ARDS lung, restoring normal C_RS_ and EELV. This approach would lower MP_rs_ at any given ventilator setting.

## 1. Introduction

**Figure 1:**
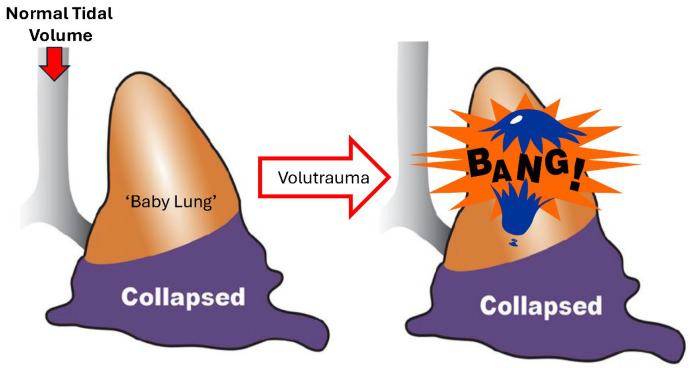
The current hypothesis for the engine that drives ventilator-induced lung injury (VILI). Suppose a large tidal volume (V_T_) is delivered to an acutely injured lung with significant collapse and loss of functional residual capacity/end expiratory lung volume (FRC/EELV). In that case, the remaining normal tissue, known as the ‘baby’ lung, will pop like a balloon [4].

The acute respiratory distress syndrome (ARDS) remains a serious medical problem, with supportive care through mechanical ventilation as the primary treatment [1]. Incorrectly set mechanical ventilation is a known cause of secondary ventilator-induced lung injury (VILI), significantly increasing ARDS-related death [2]. For more than twenty years, researchers have been working to understand the mechanisms of VILI and develop ways to mitigate it [3]. The current standard treatment for ARDS using the ARDS Network (ARDSNet) low tidal volume (LV_T_) ventilation strategy is based on the idea that overdistension of healthy lung tissue, called the ‘baby’ lung, with high V_T_ and high plateau airway pressures (P_plat_), is the leading cause of progressive VILI (Figure 1) [3].

This persistent mortality likely reflects the heterogeneity of the ARDS syndrome; patients differ in biology and anatomy (e.g., focal vs. non-focal morphology, low vs. high recruitability, hyper- vs. hypo-inflammatory phenotypes) and therefore respond variably to a one-size-fits-all strategy, as well as non-ventilatory drivers of death (sepsis, shock, multi-organ failure) and uneven adherence to protective targets. Thus, the issue may be less that LV_T_ is ineffective and more that it is insufficiently targeted to phenotype [5,6]. A significant limitation of the LV_T_ strategy is that it employs a standard 6 mL/kg V_T_ without accounting for the extent of lung damage. Amato et al. demonstrated that driving pressure (ΔP), which uses respiratory system compliance (C_RS_) in the equation (ΔP = V_T_/C_RS_), was a better predictor of ARDS outcomes than V_T_ size alone [7].

**Figure 2:**
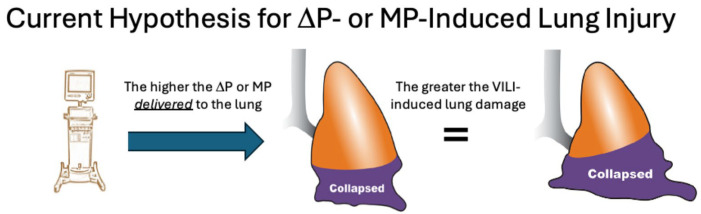
The current hypothesis is that the higher the delivered mechanical power (MP) or driving pressure (P) delivered to the acutely injured lung, the greater the ventilator-induced lung injury (VILI) and the poorer the outcomes.

*Driving pressure and Mechanical Power (MP)*: Both ΔP [7] and MP [8] are recognized as biomarkers of VILI. The core concept is straightforward: the higher the ΔP or MP delivered to the lung, the greater the tissue damage (Figure 2). Although these biomarkers reflect aspects of lung pathology, they do not directly address the immediate mechanisms of VILI-induced tissue damage, which are excessive shear and tensile strain of lung tissue [9,10]. Shear strain occurs when collapsed alveolar walls are peeled apart during recruitment, and tensile strain occurs with overdistension of alveolar and alveolar duct walls. Additionally, the primary factor that increases ΔP and MP is not ventilator settings (such as V_T_, positive end-expiratory pressure (PEEP), or respiratory rate (RR)), but rather decreased end-expiratory lung volume (EELV), functional residual capacity (FRC), and C_RS_. Therefore, the relationship between ΔP, MP, and VILI suggests increased tissue strain-induced lung damage, rather than indicating that high MP or ΔP is the mechanism of tissue injury [11].

**Figure 3:**
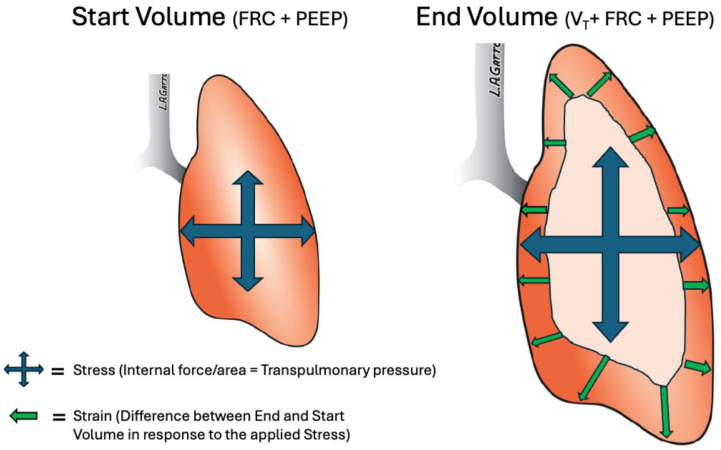
Applied stress, which for the lung during mechanical ventilation is the transpulmonary pressure, and the resulting lung tissue strain. Strain is calculated as the increase in lung volume relative to the initial volume, emphasizing the importance of a normal functional residual capacity/end-expiratory lung volume (FRC/EELV).

**Figure 4:**
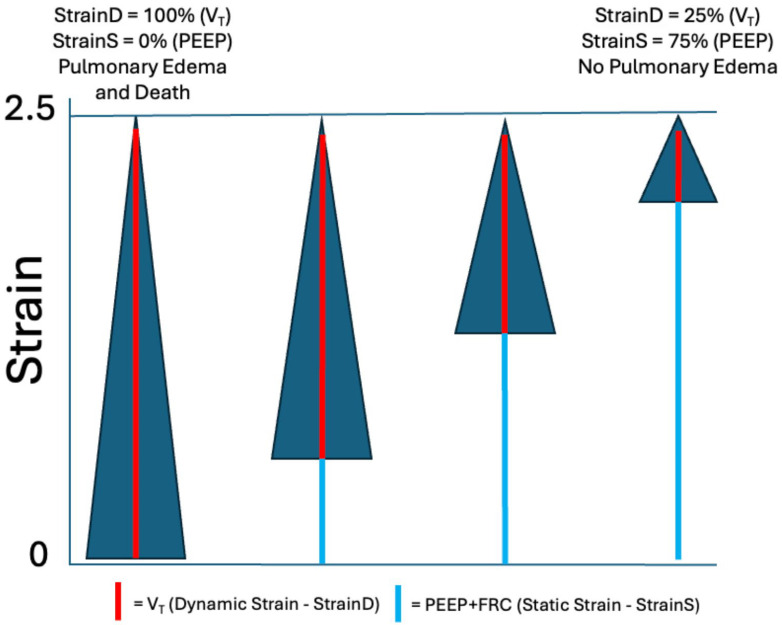
The large blue triangle represents the global strain (StrainG) in each group, which is 2.5. The roles of dynamic (StrainD; red line) and static (StrainS; light blue line) strain in StrainG–induced pulmonary edema and death are shown. Strain is measured as the number of functional residual capacity (FRC) volumes from the starting FRC. In this example, the StrainG was 2.5 FRC volumes in all four groups. StrainD was adjusted by changing the tidal volume (V_T_—red line), and StrainS by positive end-expiratory pressure (PEEP) (light blue line). If a strain of 2.5 was created solely by V_T_, it resulted in pulmonary edema and death. In contrast, the same StrainG was achieved by combining StrainD (25%) and StrainS (75%), with no edema or mortality [14].

High ΔP or MP does not directly cause VILI; rather, tissue damage results from sufficient stress (transpulmonary pressure) that leads to excessive lung tissue strain, defined as the change in volume relative to the initial volume (Figure 3). Stress without strain does not cause VILI, as shown by Dreyfuss et al. in 1988. They demonstrated that even with an inflation pressure of 45 cmH_2_O, if the chest were strapped to prevent lung volume increase (i.e., strain), VILI would be minimal [12]. It has also been found that MP was linked to mortality only when normalized to C_RS_ and EELV, underscoring the importance of lung pathophysiology in the development of VILI [13]. The normal lung does not sustain damage from high strain (2 to 2.5 times the EELV) as long as the strain remains relatively static (Figure 4) [14]. This suggests that overdistension (OD) caused by high static strain does not harm the ‘baby’ lung, which is small but normal [15]. However, the ‘baby’ lung can be injured by high dynamic strain [14].

**Figure 5:**
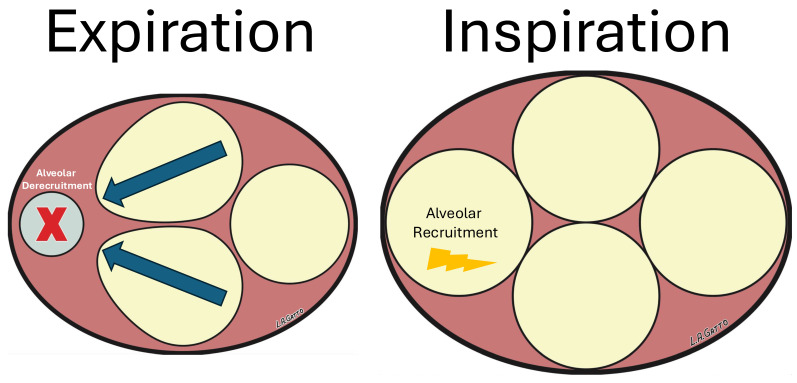
A model of repetitive alveolar recruitment and collapse (RACE) during tidal ventilation. The model consists of four open ‘alveoli’ (yellow circles) at Inspiration (Inflation) and Expiration (Deflation). We simulated regional surfactant deactivation in one ‘alveoli’ such that it collapses at expiration (red X) and is reinflated on every inspiration. Additionally, the walls of adjacent recruited alveoli are interconnected with those in collapsed alveoli, and thus they overdistend during deflation (blue arrows). Consequently, RACE causes excessive shear and tensile strain, which can dissipate concentrated energy (lightning bolt) and lead to tissue damage.

**Figure 6:**
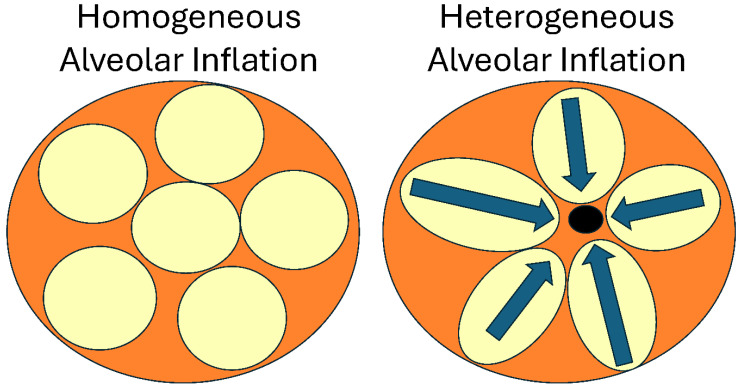
The large orange circle depicts the lung. During normal homogeneous alveolar inflation, the stress of the applied transpulmonary pressure is distributed equally among all alveoli (small yellow balls) with minimal energy dissipation or excessive strain. Acute lung injury causes regional alveolar collapse (black circle), which acts as a stress multiplier, concentrating stress in adjacent open alveoli whose walls are connected to those that collapse, leading to overdistension, energy dissipation, and excessive shear strain (blue arrows), resulting in tissue damage [4].

*Excessive lung tissue strain causes VILI*: A hallmark of ARDS pathophysiology is surfactant dysfunction, leading to alveolar instability and derecruitment [16]. This results in repeated alveolar collapse and expansion (RACE) (Figure 5) and regional collapse, which generate stress multipliers that overdistend nearby healthy tissues (Figure 6) [17,18,19,20]. RACE causes excessive shear strain (atelectrauma), while overdistension causes excessive tensile strain (volutrauma), both of which directly damage tissue. Global or Macro-lung strain (StrainG) can be measured if the FRC or EELV is known. StrainG is calculated by combining dynamic strain (StrainD = V_T_/FRC) and static strain (StrainS = PEEP volume/FRC) [14]. Due to the importance of this topic as a VILI mechanism, a detailed analysis of StrainS and StrainD is found below in the Section 4.

**Figure 7:**
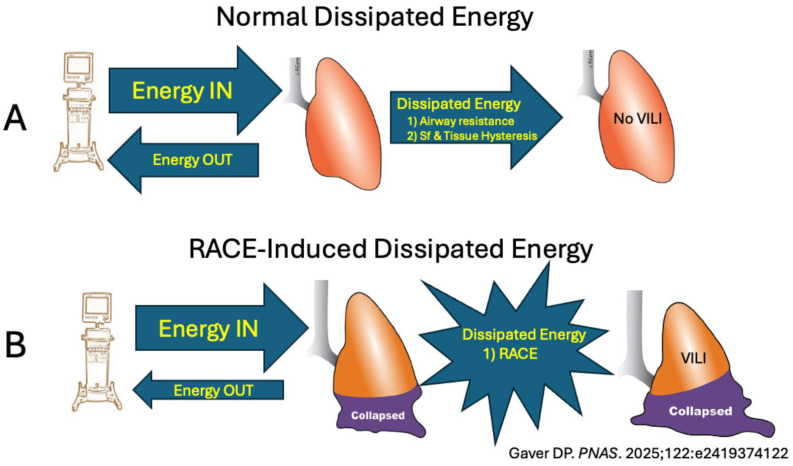
(**A**) Normal Dissipated Energy: Energy or power delivered to the lung by the mechanical ventilator cannot cause ventilator-induced lung injury (VILI) unless it is dissipated into the tissues and not returned during exhalation. Some energy is dissipated in the normal lung during each breath due to resistance to gas flow in the airways, pulmonary surfactant (Sf), and tissue hysteresis. This dissipated energy is normal and does not cause tissue damage. (**B**) RACE-Induced Dissipated Energy: Acute lung injury causes regional alveolar collapse and instability, leading to repetitive alveolar collapse and expansion (RACE). Recent work has shown that alveolar recruitment with each breath significantly increases dissipated energy and is directly associated with lung injury [21].

*Energy dissipation, not delivery, causes VILI*: High power delivered to the lung cannot cause tissue damage if all of the power is expelled during exhalation. The power must be dissipated into the tissue and not return during exhalation, or it can not cause damage. Even in a normal lung, a percentage of the power or energy delivered with each breath is lost due to airway resistance, surfactant, and tissue hysteresis, which are distributed throughout the lung [21]. This dissipated energy does not cause tissue damage or VILI (Figure 7A). However, acute lung injury deactivates pulmonary surfactant, leading to alveolar instability and RACE. Without a functioning surfactant system, excess energy is dissipated focally in regions where alveolar walls collapse, and this regional shear strain has been shown to cause VILI (Figure 7B). The dissipation of energy caused by excessive shear and tensile strain is the mechanism of VILI in the microenvironment.

In this Perspective Paper, we propose that reducing VILI-related lung tissue damage involves addressing its causes, specifically excessive shear (atelectrauma) and tensile (volutrauma) strain within the microenvironment (alveoli and alveolar ducts). ΔP and MP reflect changes in lung pathophysiology and can serve as valuable biomarkers of impending VILI when normalized to C_RS_ and EELV; however, they are indicators of disease severity rather than direct causes of injury.

## 2. Mechanical Power, Driving Pressure, and Ventilator-Induced Lung Injury (VILI)

From a bioengineering perspective, the lung can be viewed as a container with a specific limit on the amount of strain (change in volume from the initial volume) it can tolerate before stress failure happens. A healthy, uniform lung does not expand in the same way as an acutely injured, heterogeneous lung. The normally ventilated lung can endure high levels of strain without damage. For instance, athletes can generate a V_T_ of approximately 3.0 L without causing stress failure [22], while significantly less strain can harm the heterogeneously ventilated ARDS lung. Therefore, the size of the V_T_ alone does not determine VILI; both the volume and the underlying lung pathology affect the risk of stress failure. Tidal volume size and lung pathophysiology, measured as C_RS_, are factors in calculating driving pressure (ΔP = V_T_/C_RS_), which is strongly associated with increased mortality [7]. Numerous studies have shown that ΔP is a much better predictor of injurious ventilation strategies than V_T_ size alone [23,24,25].

Recently, MP delivered to the lungs has been proposed as a more effective approach to setting ventilator parameters and strategies that could reduce the risk of VILI [8,10,26]. All factors involved in mechanical ventilation contribute to the energy load on the lungs. When measured per unit time, energy reflects the MP supplied during inspiration. Mechanical power is typically calculated by multiplying each component of the motion equation by the change in lung volume (ΔV) and RR (EL_RS_ = respiratory system elastance, Raw = airway resistance, I:E = inspiratory/expiratory ratio, PEEP = positive-end expiratory pressure) [27]:

MP_rs_ = RR × {ΔV^2^∙[1/2∙EL_RS_ + RR∙(1 + I:E)/60∙I:E∙R_aw_] + ΔV∙PEEP} (1)

Numerous studies have demonstrated that high MP correlates with worse outcomes [10,27,28,29]. However, it also revealed that ΔP and RR are equally effective at predicting outcomes and are much easier to measure at the bedside [27]. Others have questioned the validity of MP as the primary mechanism of VILI [30]. The current clinical approach is to adjust ventilator settings (RR, I:E, PEEP, V_T_) to minimize MP (Equation (1)). This concept is based on the idea of a V_T_-PEEP ‘safe region’ where no lung damage occurs, which heavily depends on lung pathophysiology—specifically, RACE and stress multipliers [31]. The more severe the lung damage and the smaller the EELV, the smaller the ‘safe region’.

The role of MP in VILI is not universally accepted. A study showed no significant difference in lung injury between high and low MP over 48 h in a porcine model [32]. Coppola et al. demonstrated, through a retrospective analysis of ARDS cases from seven published studies, that there is no causal relationship between MP and mortality [13]. Dianti et al. demonstrated that MP did not provide better information regarding the risk of death from VILI compared to V_T_ size or ΔP in a meta-regression involving adults with ARDS [33]. A pooled database of ARDS patients from six randomized trials of lung-protective ventilation and a large observational cohort found that higher MP was associated with increased mortality. Still, it was no better than RR or ΔP at predicting outcomes. Instead, MP normalized to C_RS_, and EELV was associated with mortality, highlighting the importance of lung pathophysiology in the development of VILI [13,27].

In summary, the MP is the energy per unit time delivered to the respiratory system, generated by the applied V_T_, PEEP, RR, and flow. This review of lung stress and strain during mechanical ventilation has shown that the ΔP and MP can be manipulated by components of the mechanical breath (V_T_, Pplat, PEEP) and are magnified by changes in lung pathophysiology (C_RS_, EL_RS_). Still, they are not a direct measure of lung strain caused by the delivered mechanical breath. Although the ΔP is a metric indexed by the size of the lung, being inversely proportional to C_RS_, and it correlates with patient outcomes, it is essential to remember that a high ΔP is not the injury mechanism itself. Injury occurs only if high ΔP causes excessive strain [10].

## 3. Emerging Concepts in the Mechanisms of VILI-Induced Lung Damage

The ARDS, identified as a syndrome in 1967, causes heterogeneous injury characterized by normal, unstable, and edema-filled tissue spread throughout the lung. Since the Acute Respiratory Management in ARDS (ARMA) study in 2000, it has been understood that improperly set mechanical ventilation for patients with ARDS can unintentionally increase mortality [34]. This VILI was believed to occur from higher V_T_ and P_plat_, which overdistended the remaining normal tissue, known as the ‘baby lung’. The study also showed that ARDS-related mortality could be reduced if V_T_ was set at or below 6 mL/kg and P_plat_ at or below 30 cmH_2_O. The early enthusiasm for the low V_T_ protective ventilation strategy waned over the following decade, as meta-, statistical-, and observational analyses indicated that ARDS-related mortality remained unchanged or even increased compared to the ARMA study [6,35].

The hypothesis behind the ARMA protocol was that OD of the remaining ‘baby lung’ was the mechanism driving VILI (Figure 1). However, there are some issues with this hypothesis, including the following: (1) there is no direct evidence definitively linking OD with increased mortality; (2) animal studies show that high static airway pressure in normal lungs with strain levels above 2 do not cause lung injury [36]; (3) allowing the lung to remain collapsed is problematic since long-term atelectasis causes lung tissue injury [37]; and (4) low volume/pressure ventilation promoting progressive lung collapse drives the lung into the VILI-Vortex, reducing the amount of tissue exposed to ventilation stress [38].

**Figure 8:**
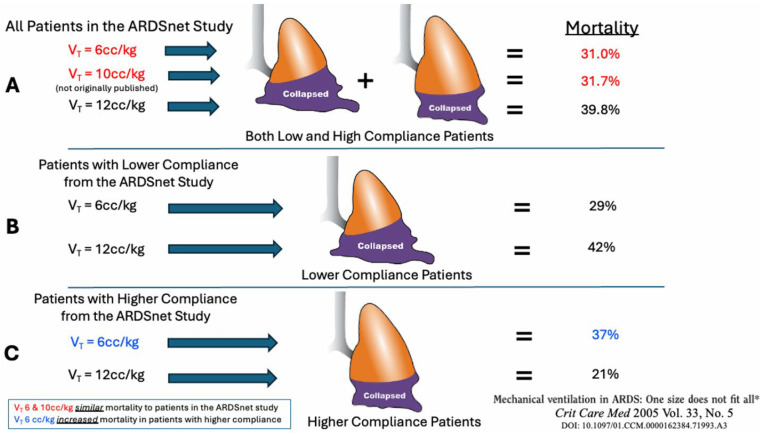
A retrospective analysis was conducted to examine the impact of tidal volume (V_T_) on mortality in patients with low and high respiratory system compliance (C_RS_), using data from the ARDS Network (ARDSnet) study. (**A**) A group of patients (2587) who were excluded from the ARDSnet study (not orginally published) but had similar recorded data received standard-of-care ventilation at the time, which was 10 mL/kg: All patients, regardless of C_RS_, who were ventilated with a V_T_ of 10 mL/kg, showed similar mortality rates as those with 6 cc/kg (red). (**B**) Reanalyzing just the patients in the ARDSnet study, those with lower C_RS_, a V_T_ of 6 mL/kg, significantly reduced mortality compared to those with higher C_RS_. (**C**) However, in patients with higher C_RS_, the *low V_T_ of 6 cc/kg increased mortality* (*blue*) [39].

In 2005, Deans analyzed data from the ARDSNet ARMA study for a subgroup analysis [39]. Patients were divided into high- and low-compliance groups, and mortality rates were examined. The results were quite revealing. In patients with low lung compliance, low V_T_ (6 mL/kg) decreased mortality, and high V_T_ (12 mL/kg) increased mortality, as expected. However, in patients with higher lung compliance, low V_T_ was linked to higher mortality, while high V_T_ was associated with lower mortality (Figure 8). Clearly, a one-size-fits-all V_T_ is not lung-protective or harmful; instead, lung compliance, which indicates lung size and health, plays a key role in lung injury or protection.

As mentioned earlier, the initial hypothesis behind the ARMA protocol was that OD of the remaining ‘baby lung’ was the primary cause of VILI when ventilating an ARDS lung with a relatively normal-sized V_T_ (Figure 1) [15]. However, this hypothesis does not align with recent studies on VILI mechanisms, as follows: (1) OD-induced tissue injury occurs not in normal tissue (i.e., ‘baby’ lung) but in areas adjacent to regional collapse, causing stress multipliers (Figure 6) [40]. Stress multipliers can lead to regional excessive strain even when the macro whole-lung or global strain levels are within safe limits [17,18,41,42]. (2) High static strain (2.0–2.5), produced with low V_T_ and high PEEP, does not cause damage to normal lung tissue, whereas the same dynamic strain (high V_T_ and low PEEP) causes severe lung injury and death in a 54 h porcine VILI model (Figure 4).

As discussed in the Introduction, strain is defined as the change in lung volume from its initial volume (FRC/EELV) plus PEEP volume. This indicates that high dynamic strain, not OD, is the primary mechanism behind VILI in the ‘baby’ lung (Figure 4) [36]. Alveolar instability (i.e., RACE) directly causes damage to lung tissue [43]. (3) During exercise, V_T_ can exceed 2 L in a normal lung without causing damage [22] and (4) in lungs with heterogeneous injury compared to healthy lungs, tissue damage does not occur in normal tissue; instead, it spreads concentrically from stress multipliers caused by collapsed or edema-filled tissue (Figure 6 Black Dot), at a lower strain threshold (1.29) than in normal lungs (2.0) [44] in a poor get poorer fashion [45]. Because these collapsed regions are small and not visible with standard bedside imaging, they are called ‘hidden microatelectasis’ and have been shown to increase vulnerability to VILI [46].

## 4. VILI Mechanisms: Global Static and Dynamic Strain

Protti et al. conducted a study on normal pigs, combining animals from previous research with new experiments ventilated for 54 h, during which they varied the ratio of static to dynamic strain (Figure 4) [14]. The lower and upper limits of inspiratory capacity were identified using computed tomography (CT). Global strain (StrainG) was calculated by combining dynamic strain (StrainD = V_T_/FRC) and static strain (StrainS = PEEP volume/FRC).

StrainG = SrainD + StrainS

Experiments were carried out with StrainD ranging from 18% to 100% of StrainG. The Strain_G_ range was adjusted from 0.45 to 5.56, with an inspiratory capacity of 2–3 times the FRC.

Inspiratory capacity was measured using CT as the difference between TLC and FRC, with the upper and lower limits determined from the volume–pressure curve. Animals were divided into three groups based on V_T_ and PEEP combinations to target inspiratory volume as ‘Below,’ ‘Within,’ or ‘Above’ inspiratory capacity.

The energy load on the lung includes a static component composed of the PEEP and the lung volume generated by PEEP.

Static energy load = (PEEP × PEEP volume)/2

and a dynamic component caused by ΔP:

Dynamic energy load = (PEEP + Peak pressure) × V_T_/2

The cyclic energy load is the tidal change in lung volume, with energy calculated as the pressure applied multiplied by the volume change (P × dV) during inspiration. Thus, PEEP does not contribute to the cyclic energy load since there is no change in volume (dV = 0). The goal of the study was to determine whether there is a threshold for lung-volume distortion or energy load that causes VILI [14].

This was achieved by testing different tidal volumes (StrainD), (P_TP_) (P_TP_ = dynamic stress), and PEEP levels (static strain and static stress), allowing them to define the interaction between lung volume limits measured by CT, inspiratory volumes (V_T_ + PEEP), and the dynamic and static energy involved in lung injury development (Figure 4) [14].

Results showed that pigs ‘Below’ inspiratory capacity did not develop edema, whereas those ‘Within’ developed edema with a 52% mortality; the amount of edema correlated with StrainD. In the ‘Above’ group, pulmonary edema did not develop, but 66% died rapidly. If V_T_ had not been lowered, adding PEEP would have increased mortality. It was concluded that VILI did not occur within the lower limits of inspiratory capacity; however, within these limits, VILI could happen depending on the severity of StrainD. Stress failure of tissues occurs above the inspiratory capacity, and PEEP is protective when combined with reducing V_T_; otherwise, it has no effect or is harmful [14].

Although the results of this study are fascinating, they should be viewed in the context of differences between the ARDS lung and a normal lung: the ventilation of a normal lung is even, whereas that of an ARDS lung is uneven. Heterogeneity is characterized by regional areas of alveolar collapse and edema-filled tissue, which cause RACE and create stress multipliers. RACE differs from normal StrainD (V_T_/FRC) because loss of pulmonary surfactant leads to alveolar collapse during exhalation, which reopens during inspiration, significantly increasing and concentrating energy dissipation (Figure 7) [21]. This can occur within the safe limits of whole-lung strain, depending on the severity of surfactant dysfunction-induced loss of EELV and decrease in C_RS_.

These findings were supported by Pistillo et al., who hypothesized that, if patients were ventilated with the same respiratory system mechanical power (MP_RS_), the risk of VILI would depend on the size of the remaining aerated lung (ergotrauma) [47]. They calculated both the specific mechanical power (SMP = MP_RS_/C_RS_) and the specific lung mechanical power (SLMP), which is calculated as the ratio of transpulmonary mechanical power (MP_TP_) to EELV (SLMP = MP_TP_/EELV). Stress was calculated as the P_TP_ at peak inspiration. Strain was calculated using tidal volume (V_T_) and EELV (Strain = V_T_/EELV), and atelectrauma as the difference between expiration and inspiration in the nonaerated lung (i.e., RACE), measured by computer tomography (CT).

SLMP increased linearly as patients progressed from mild to severe ARDS, possibly due to EELV loss. They found that mechanical power correlates with stress, strain, and atelectrauma only when normalized to a C_RS_ or EELV in patients with ARDS. Both SLMP and SMP were highly correlated with stress, strain, and atelectrauma. This makes sense because VILI is not solely due to the mechanical energy delivered to the lung during inspiration. Instead, it depends on the size and pathophysiology of the lung receiving the energy. They concluded that patients ventilated with similar mechanical power have different risks of developing VILI, based on the severity of ARDS.

How do these findings enhance the understanding of the role of ΔP and MP in VILI? The EELV, FRC, EL_RS_, and C_RS_ are highly variable in ARDS patients and are essential when calculating ΔP and MP. This is why the Amato study [7] showed that ΔP (V_T_/C_RS_) was more effective at stratifying risk in ARDS patients than V_T_ alone, a method of setting mechanical ventilation that overlooks lung size and pathophysiology, such as EELV, FRC, C_RS_, and EL_RS_.

*Stress and Strain Normalized to Lung Size and Compliance*: Chiumello et al. measured lung stress and strain using titrated V_T_ and PEEP in four patient groups: (1) post-surgery, (2) with medical diseases, (3) with acute lung injury, and (4) with ARDS [48]. Their main finding is that ideal body weight (IBW)-based V_T_ and airway plateau pressure are inadequate proxies for lung stress and strain. They observed significant variability in (1) *lung stress* (P_TP_), caused by differences in lung elastance (EL) relative to respiratory systems elastance (EL_RS_), calculated as EL/(EL + E_CW_) ratio (ranging from 0.33 to 0.92 in ARDS patients and 0.36 to 0.95 in surgical and medical patients), and (2) *lung strain*, which depends on functional residual capacity (FRC)—the smaller the FRC, the greater the strain—for any given airway pressure across all four groups.

The specific lung elastance (EL_spec_) is the (P_TP_) at which the FRC doubles. EL_spec_ remained nearly constant across all four groups at 13.5 cm H_2_O, indicating that a similar change in (P_TP_) (ΔP_TP_) produces a comparable *fractional change* in FRC in both normal and ARDS lungs. In other words, it reflects the stress-to-strain ratio. Therefore, for a similar P_TP_, the normal lung might increase from an FRC of 3 L to 6 L, while the ARDS lung might increase from 0.5 L to 1 L.

**Figure 9:**
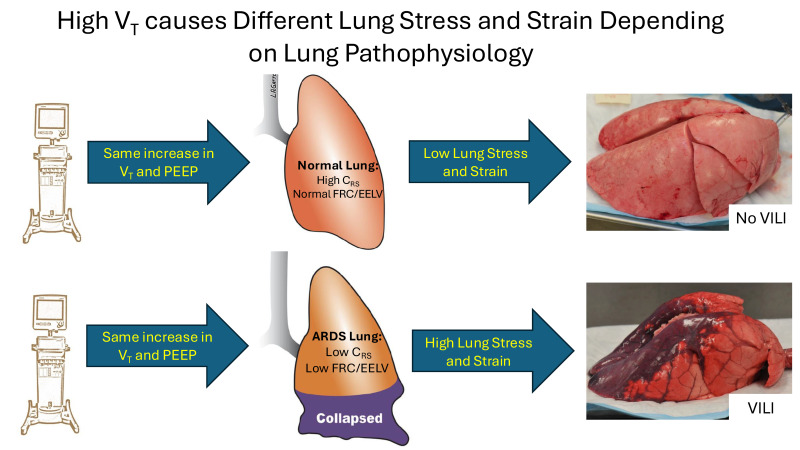
It is not just the size of the tidal volume (V_T_) and positive end-expiratory pressure (PEEP) that cause excessive lung stress and strain, but rather the size (functional residual capacity—FRC or end-expiratory lung volume—EELV) and compliance (C_RS_) of the lung into which the mechanical breath is delivered. Therefore, identical driving pressure and mechanical power can be either protective or harmful to the lungs. **Top**: Increasing V_T_ and PEEP in a normal lung with high C_RS_ and normal EELV generates only a slight increase in lung stress and strain, and thus no ventilator-induced lung injury (VILI). **Bottom**: A similar rise in V_T_ and PEEP in an acutely injured lung with low C_RS_ and EELV can cause damaging stresses and strain, resulting in VILI [48].

**Figure 10:**
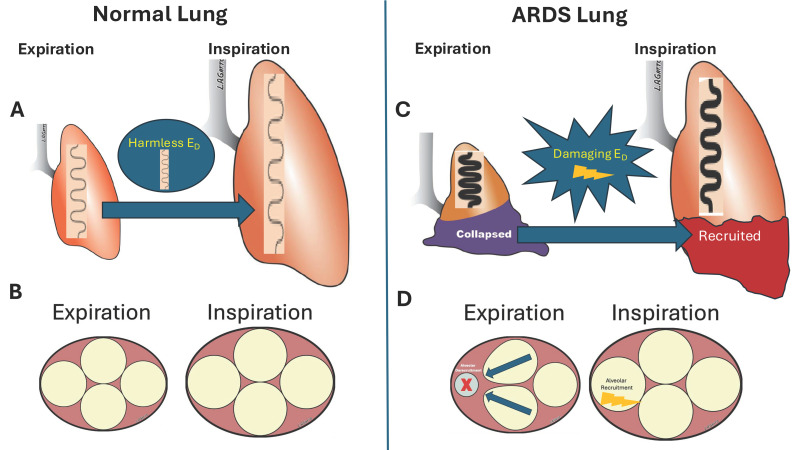
(**A**) In the normally inflated, homogeneous lung, energy is dissipated harmlessly (E_D_) through airway resistance, viscoelastic expansion of surfactant, and tissue stretch during each breath (i.e., the thin spring within the lung). (**B**) At the alveolar level, alveoli (yellow balls) do not collapse during expiration, and the E_D_ is distributed equally throughout the lung. (**C**) Acute lung injury deactivates pulmonary surfactant, thereby significantly increasing lung recoil and shortening the collapse time constants (i.e., the thick spring within the lung). This results in regional alveolar instability (repetitive alveolar collapse and expansion—RACE) and regional alveolar collapse. A portion of the collapsed tissue (purple) recruits during inspiration (red) and re-collapses at expiration (RACE), resulting in a highly focused E_D_ (lightning bolt). (**D**) At expiration, one ‘alveolus’ (yellow ball) collapses (red X). During inflation, highly concentrated E_D_ occurs as the alveolar walls peel open. In addition, the collapsed alveolus during deflation (red X) acts as a stress multiplier, leading to overdistension and excessive tensile strain in the adjacent open alveoli (blue arrows) [4].

Since lung elastance (EL) can vary greatly, the fraction of airway pressure that distends the lung (ΔP_TP_) can differ nearly threefold. The high variability in FRC and EL_RS_ also affects the absolute MP calculation and may not reflect the actual power delivered to the lung. Similar to ΔP, an MP safe for a lung with high FRC and low EL may not be safe for a lung with low FRC and high EL_RS_. This further demonstrates that strain size results from a combination of the applied stress (ΔP_TP_) and lung pathophysiology. They conclude that a safe threshold for ‘harmful’ stress and strain does not exist unless FRC and EL_spec_ are known (Figure 9). These studies show that lung tissue injury is caused not by the size of ΔP or MP, but by the excessive strain they can create. Whole lung dynamic strain (StrainD) is much more damaging than high static strain (StrainS) in a healthy lung [14]. Even if StrainD is kept at safe levels for healthy tissue, regional RACE and stress multipliers can still lead to tissue damage in acutely injured lungs (Figure 10C,D) [17,18,19,20].

To determine if the strain magnitude causes VILI, it should be normalized to EL and EELV. Since regional collapse and RACE are the main factors for excessive strain and VILI, our ventilator strategies should focus on eliminating these lesions. Currently, we are limited in setting ventilator parameters to prevent further damage to a regionally injured, heterogeneous lung. The only way to reduce ΔP and MP is to lower airway pressures, volumes, and rates. Wouldn’t a better strategy be to break these constraints by increasing FRC/EELV and improving EL_RS_ and C_RS_ for a given set of ventilator settings?

## 5. Treat the Lung, Not the Ventilator

**Figure 11:**
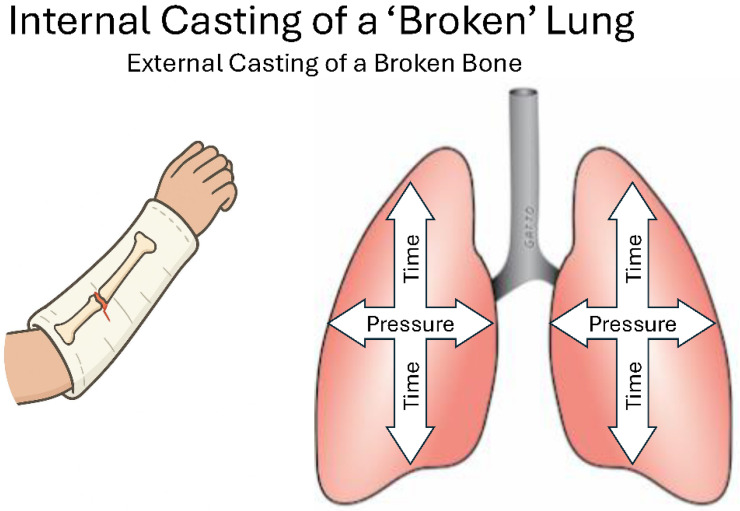
The optimal lung-protective strategy is similar to applying an external cast to a broken arm until it heals. The cast stabilizes the bone, allowing it to heal in its natural position. To protect the acutely injured, heterogeneously ventilated lung from ventilator-induced lung injury (VILI), an internal cast can be used by combining longer inspiratory times with brief expiratory durations and appropriate airway pressures. The goal is to keep the lung, which depends on time and pressure, in its normal anatomical position until healing occurs [4].

Instead of adjusting ventilator settings to decrease MP in a heterogeneously ventilated lung, it is better to address the underlying pathophysiologic issues: abnormal C_RS_, EL_RS_, and low EELV. This can be achieved by stabilizing the lung internally with appropriate airway pressures and timing, similar to stabilizing a broken arm with an external cast (Figure 11) [4,49]. This approach lowers the ΔP or MP delivered with each breath by increasing C_RS_ and decreasing EL_RS_, as shown in Equation (1), thereby expanding the V_T_-PEEP ‘safe region.’ However, stabilizing and reopening the acutely injured lung to normalize C_RS_, EL_RS_, and EELV remains a challenging task.

**Figure 12:**
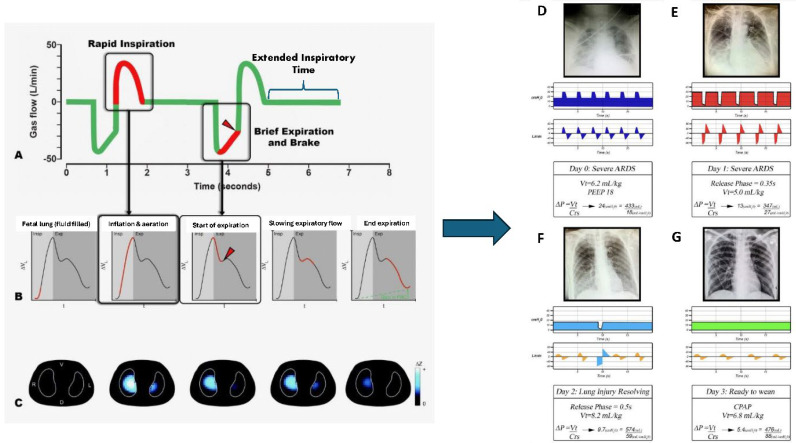
A novel ventilator strategy for patients with acute respiratory distress syndrome (ARDS) using a gradual lung recruitment method utilizing an extended inspiratory and very brief expiratory time (**A**), which is similar to the method a newborn uses to open their fluid fill lung at birth (**B**,**C**): (**A**) A mechanical breath airway gas flow/time curve (green) using the airway pressure release ventilation (APRV) mode. Note the extended inspiratory time and brief expiratory duration (**B**). A full-term newborn’s spontaneous first breath volume/time curve. (**C**) The newborn’s corresponding lung volumes were measured by electrical impedance tomography (EIT). (**B**,**C**) *Fetal lung (fluid-filled)*: The newborn lung is fluid-filled and airless ((**C**) black, far left). *Inflation and Aeration*: The deep breath to start the ‘cry’ ((**B**) red) partially inflates both lungs ((**C**) blue). *Start of Expiration*: Closing the glottis acts as an expiratory brake, slowing expiratory gas flow ((**B**) red arrow) and reducing lung volume loss ((**C**) blue). *End of expiration*: A small functional residual capacity (FRC) has been established at the end of the first breath, far right ((**C**) blue), which grows with each subsequent breath [54]. (**A**) Similar to the newborn, the APRV mechanical breath works by opening the lung and preventing re-collapse using an ‘inflate and brake’ ratcheting method. Rapid Inspiration from the termination of expiration acts similarly to the newborn’s deep inspiration to begin a cry (red) to recruit a small amount of tissue with each breath. Lung recruitment is enhanced by an extended inspiratory time, which allows lung tissue with long opening time constants to open. Expiratory duration is very brief (≤0.5 s, red arrow) and acts as a brake to prevent the recollapse of the newly recruited tissue. (**D**–**G**) Progressive lung recruitment of an ARDS patient’s lung using the APRV mode. (**D**) On ***Day 0***, an ARDS patient with a partially collapsed lung (whiteout X-ray) was on the ARDSnet ventilation strategy. Positive end expiratory pressure (PEEP, 18 cmH_2_O) and driving pressure (ΔP, 24 cmH_2_O) are high, and respiratory system compliance (C_RS_, 18 mL/cmH_2_O) is low. (**E**) On ***Day 1*** after switching to the APRV mode, the lungs begin to open (X-ray less white). Expiratory time is very brief (release phase = 0.35 s) to prevent recollapse. C_RS_ has increased, whereas ΔP has decreased. (**F**) On ***Day 2***, most of the lung volume has been restored, and the number of release phases has been reduced, as the patient is providing most of their minute ventilation (M_VE_) via spontaneous breathing (gold waveforms). (**G**) On ***Day 3***, the lung is fully recruited with normal C_RS_ and ΔP. Reprinted with permission of the American Thoracic Society. Copyright © 2025 American Thoracic Society. All rights reserved. Cite: Tingay, D.G.; Farrell, O.; Thomson, J.; Perkins, E.J.; Pereira-Fantini, P.M.; Waldmann, A.D.; Rüegger, C.; Adler, A.; Davis, P.G.; Frerichs, I. Imaging the Respiratory Transition at Birth: Unraveling the Complexities of the First Breaths of Life. *Am. J. Respir. Crit. Care Med.* **2021**, *204*, 82–91 [54]. *The American Journal of Respiratory and Critical Care Medicine* is an official journal of the American Thoracic Society. (**D**–**G**) Reproduced from Nieman, G.; Kollisch-Singule, M.; Ramcharran, H.; Satalin, J.; Blair, S.; Gatto, L.A.; Andrews, P.; Ghosh, A.; Kaczka, D.W.; Gaver, D.; et al. Unshrinking the baby lung to calm the VILI Vortex. *Crit. Care* **2022**, *26*, 242, https://doi.org/10.1186/s13054-022-04105-x, under the Creative Commons Attribution-NonCommercial (CC BY-NC 4.0) license (https://creativecommons.org/licenses/by-nc/4.0/) [57].

The two methods tested in randomized controlled clinical trials to reopen the ARDS lung—high-frequency oscillatory ventilation [50,51] and aggressive recruitment maneuvers over a few minutes—have failed to establish durable lung inflation or reduce mortality [52]. A new concept is to ‘ratchet’ the lung open gradually over hours or days, depending on the severity of lung disease [53]. This innovative ventilation strategy makes physiological sense because the lung behaves as a viscoelastic system, and ARDS makes it even more time- and pressure-dependent [4]. This means it takes longer for the lung to open and less time to recollapse at any given airway pressure. Therefore, extended inspiration time will recruit a small number of alveoli with each breath, and a sufficiently brief expiratory duration will prevent recollapse (Figure 12A). As a result, alveoli will be ‘ratcheted’ open slowly with each breath over hours or days based on established physiological principles.

The gradual ‘ratcheting’ open of lung tissue during mechanical ventilation has its roots in evolutionary biology. Tingay et al. demonstrated that full-term newborn infants open their fluid-filled lungs at birth through this breath-by-breath ‘ratchet’ mechanism [54]. Figure 12B shows a volume/time curve, and Figure 12C illustrates the resulting electrical impedance tomography (EIT) lung volume of the newborn’s first breath. The lung initially has no gas volume (black EIT). The next stage involves *inflation* and *aeration* as the infant fills their lungs to begin a ‘cry’ (Figure 12B, red). This process resembles the *rapid inspiration* of the lung during mechanical ventilation (Figure 12A, red). The innovation lies in preventing the newly inflated lung tissue (Figure 12C, blue) from recollapsing. Gas volume decreases quickly at the *start of expiration.* Still, by closing the glottis and halting active expiration to *slow expiratory flow* (Figure 12B, red), a small amount of lung tissue remains open at *the end of expiration*, increasing functional residual capacity (FRC) (Figure 12C, blue).

With an acutely injured lung, mechanical breath cannot simply slow expiratory flow to prevent collapse, as the surfactant-deficient lung is now time- and pressure-dependent [4]. Instead, a *Brief Expiration* acts as a ‘brake’ to stop collapse at which point the lung is rapidly inflated (Figure 12A, red arrow) [55]. The *Extended Inspiratory Time* maintains the slow alveolar recruitment of viscoelastic tissue during each breath (Figure 12A) [56].

Although the hypothesis is unproven in the lung, the rapid change in gas flow direction from expiration to reinflation may act as a ‘gas-hammer’ to further increase alveolar recruitment with each breath. During the brief expiratory phase, gas flows quickly out of the lung and are abruptly stopped, and the lung is rapidly reinflated (Figure 12A). Thus, the inspiratory and expiratory gas flows ‘crash’ into each other, generating considerable energy dissipation. It has been shown that reversing gas flow in pipes can produce a transient pressure surge that can generate stress amplification, similar but less intense than the ‘water hammer’ with liquids. Work from natural gas pipelines supports the idea that rapid gas flow reversal induces transient overpressures and stress amplifications. The role of the ‘gas-hammer’ in lung recruitment is an area for future research.

An example of how gradual ‘ratcheting’ affects lung inflation via chest radiograph (CXR), V_T_, C_RS_, and ΔP over three days (Figure 12D–G). On ***Day 0***, the patient is on ARDSNet low V_T_ ventilation with high PEEP. The lungs are collapsed (white CXR), and ΔP is high because C_RS_ is very low. On ***Day 1***, the ventilator strategy is adjusted to include longer inspiratory times and brief expiratory durations, aiming to open and prevent re-collapse of lung tissue with each breath. The lung begins to open, V_T_ is less than 6 mL/kg, and ΔP improves because C_RS_ has increased. On ***Day 2***, the number of expiratory releases decreases, as the patient generates most of their minute ventilation (M_VE_) through spontaneous breathing (gold waveforms). The lung is nearly fully recruited, V_T_ and C_RS_ have increased, and ΔP is within a safe range. By ***Day 3***, the lung is fully recruited, all M_VE_ is patient-generated, and C_RS_ and ΔP return to normal [57].

## 6. Summary

Elevated MP and ΔP are useful biomarkers of potential VILI when they are normalized to FRC/EELV and C_RS_. It is not the power or energy delivered to the lung that causes tissue damage, but rather the energy dissipated and not returned during exhalation that causes injury. A recent study has shown that the amount of energy dissipated during alveolar recruitment directly correlates with the severity of lung injury [21]. The stress applied to the lung during inspiration strains the alveoli and alveolar ducts. Excessive shear (atelectrauma) and tensile (volutrauma) strains lead to tissue damage. Shear strain can be minimized by preventing alveolar collapse during expiration. Tensile strain can be reduced by eliminating the stress multipliers caused by regional lung collapse.

What does all of this mean in relation to protective mechanical ventilation? It demonstrates that a lung-protective strategy that does not normalize EELV is likely to fail. Modifying ventilator settings to lower high MP and ΔP is constrained to ventilating a heterogeneously injured lung and does not address the root cause of VILI by eliminating the pathophysiology. The ‘catch 22’ is that strategies that attempt to reestablish EELV over seconds or minutes rapidly have failed [52]. The best way to lower high MP and ΔP that can cause strain-induced damage is not by reducing ventilator settings (V_T_, RR, PEEP, I:E), but rather stabilizing and gradually “ratcheting” open the lung, restoring normal EELV using a method similar to how full-term infants open their fluid-filled lungs at birth (Figure 12) [53,54].

## Data Availability

Not applicable.
